# Hydrogen Adsorption on Ordered and Disordered Pt–Fe and Pt–Co Alloys

**DOI:** 10.1021/acs.jpcc.4c01308

**Published:** 2024-06-29

**Authors:** Andrew Okafor, William A. Shelton, Ye Xu

**Affiliations:** † Cain Department of Chemical Engineering, Louisiana State University, Baton Rouge, Louisiana 70803, United States; ‡ Department of Physics and Astronomy, Louisiana State University, Baton Rouge, Louisiana 70803, United States

## Abstract

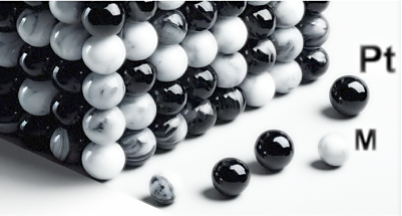

The bulk properties and surface chemical reactivity of compositionally disordered Pt–Fe and Pt–Co alloys in the *fcc* A1 phase have been investigated theoretically in comparison to the ordered alloys of the same compositions. The results are analyzed together with our previously reported findings for Pt–Ni. Nonlinear variation is observed in lattice constant, d band center, magnetic moment, and hydrogen adsorption energy across the composition range (0–100 atomic % of Pt, *x*_Pt_). The Pt 5d states are strongly perturbed by the 3d states of the base metals, leading to notable density of states above the Fermi level and residual magnetic moments at high *x*_Pt_. Surface reactivity in terms of average H adsorption energy varies continuously with composition between the monometallic Fe–Pt and Co–Pt limits, going through a maximum around *x*_Pt_ = 0.5–0.75. Close inspection reveals a significant variation in site reactivity at *x*_Pt_ < 0.75, particularly with disordered Pt–Fe alloys due in part to the inherent disparity in chemical reactivity between Fe and Pt. Furthermore, the strong interaction between Fe and Pt causes Pt-rich sites to be less reactive toward H than Pt-rich sites on disordered Pt–Ni alloy surfaces, despite less compressive strain caused. These results provide theoretical underpinnings for conceptualizing and understanding the performance of these Pt-base metal alloys in key catalytic applications and for efforts to tailor Pt-alloys as catalysts.

## 1 Introduction

Pt-based alloys have been extensively studied over the years in both thermocatalysis and electrocatalysis by researchers seeking better catalytic performance than monometallic Pt. For example, binary Pt alloys have been reported to enhance the electrochemical activity of Pt for hydrogen evolution reaction (HER) and oxygen reduction reaction (ORR). These include alloys of Pt with base metals, alkaline earth metals, and lanthanides,^1−7^ generating comparable or higher H_2_ or O_2_ current densities.

Pt–Fe, Pt–Co, and Pt–Ni form ordered face-centered tetragonal (*fct*) L1_0_ phases at a composition of PtM. Among these, PtFe has an order/disorder transition critical temperature (*T*_C_) that exceeds 1300 °C, which reflects a remarkable degree of structural stability of this intermetallic compound. It is followed by PtCo and PtNi with a *T*_C_ of over 800 and 600 °C, respectively.^8^ Sun and coworkers have shown in a series of studies that PtFe nanoparticles in the *fct* phase are chemically stable at high potentials in acidic electrolytes. The nanoparticles feature Pt-rich exteriors with improved catalytic properties, creating an overall superior electrocatalyst for ORR compared to monometallic Pt or disordered Pt–Fe.^9,10^ Similar findings have been reported by other workers for Pt–Co.^11−13^ Ordered Pt–Co nanoparticles are found to be more active for ORR than disordered ones, although there is conflicting evidence in the literature on whether ordered or disordered phases were more stable under reaction conditions.^1,13^ The enhanced catalytic properties have been explained on the basis of both electronic and geometric factors and the interplay thereof^2,14^ and how the catalysts evolve under reaction conditions forming active structures such as Pt-rich skins.^3^

Annealing precursors above the order/disorder *T*_C_ followed by rapid quenching is necessary to ensure the formation of Pt–M alloys in the *fcc* A1 phase,^1,13,15^ while annealing below the *T*_C_ or allowing slow cooling favors ordering.^16^ In many studies (e.g., Pt–Fe^17^ and Pt–Co^12,18,19^), samples annealed at temperatures well below the *T*_C_ were used to represent disordered Pt–M alloys. Although the small size of nanoparticles may lower the *T*_C_,^20^ it is doubtful whether a compositionally disordered A1 phase or instead a material containing heterogeneous microstructures was obtained in those studies. Due to a lack of consistency in the literature, the chemical and catalytic properties of disordered A1 phases of these binary alloys remain unclear.

Previously, we modeled ordered and disordered Pt–Ni alloys in the A1 phase and investigated bulk properties and surface reactivity as a function of composition.^21^ Plane-wave (PW) density function theory (DFT) calculations were performed based on large supercells generated using the SCRAPs algorithm,^22^ in comparison with Korringa–Kohn–Rostoker coherent potential approximation (KKR-CPA) calculations, which is a mean-field method that treats compositional disorder and average electronic structure on equal footing. The two methods produced bulk lattice constant and electronic structure for disordered Pt–Ni alloys in close agreement with each other. Furthermore, as H_2_ is one of the simplest molecules and is frequently used as a probe of surface reactivity,^23^ the adsorption of atomic H was compared on the (111) facets of ordered vs disordered Pt–Ni alloys. In terms of the average adsorption energy of H, the reactivity of ordered PtNi_3_, PtNi, and Pt_3_Ni was found to be similar to that of the disordered Pt–Ni alloys of the same overall compositions, i.e., Pt_0.25_Ni_0.75_, Pt_0.50_Ni_0.50_, and Pt_0.75_Ni_0.25_.

In this paper, we extend the same methodology to two other widely studied binary Pt alloys, Pt–Fe and Pt–Co. We continue to focus on the compositionally disordered alloys in the *fcc* A1 phase as a class of structurally uniform, kinetically frozen materials that can potentially be developed into tunable catalysts. Certain trends emerge when we analyze the results for the three groups of Pt-base metal alloys together. The Pt 5d states are strongly perturbed by the base metal 3d states due to spin polarization and have a significant presence above the Fermi level. Surface reactivity in terms of average H adsorption energy varies continuously between the monometallic limits, but unlike Pt–Ni, a maximum occurs at *x*_Pt_ = 0.5–0.75 for Pt–Fe and Pt–Co. Further analysis reveals a distribution of site reactivity, which is generally more pronounced for *x*_Pt_ < 0.75, particularly for Pt–Fe. Evidence of complementary strain and ligand effects is presented.

## 2 Methods

Period, spin-polarized DFT calculations were performed using the Perdew, Burke, and Ernzerhof generalized gradient functional (GGA-PBE)^24^ using plane-wave- and Green’s function-based methods.

The plane-wave (PW) calculations were performed using the Vienna ab initio simulation package (VASP).^25,26^ The core potentials were described by the projector-augmented wave (PAW) method,^27,28^ while the Kohn–Sham valence states [Co/Fe(3d4s), Pt(5p5d6s), and H(1s)] were expanded in a plane-wave basis set with a cutoff energy of 650 eV and smeared with a width of 0.2 eV using a first-order Methfessel–Paxton scheme.^29^ The convergence criterion for the electronic self-consistent field cycle was set to 10^–5^ eV. The other approach was the all-electron Korringa–Kohn–Rostoker coherent potential approximation (KKR-CPA). KKR is a Green’s function method for calculating electronic structure and total energy,^30,31^ while the CPA is a mean-field theory method for calculating the configurationally averaged Green’s function for substitutionally disordered alloys^32,33^ and including Friedel screening in metals.^34^ The atomic potentials were modeled within the atomic sphere approximation (ASA),^35^ supplemented by variationally optimized potential energy zero resulting in formation of energies matching full potential results.^36^ The valence states were expanded in a spherical harmonic basis that included angular momentum up to lmax = 4. Green’s function for each system was found using a semicircular Gauss–Chebyshev contour integration with 20 complex energy points. With both approaches, the total energy was further minimized with respect to the magnetic moment. We did not explicitly model paramagnetic states.

All ordered and disordered alloys in this study were modeled in the *fcc* crystalline structures. The ordered 1:3 and 3:1 phases (PtCo_3_, PtFe_3_, Pt_3_Co, and Pt_3_Fe) were modeled in the L1_2_ structure. The ordered PtCo and PtFe phases were modeled in a distorted L1_0_ structure with *c* = *a* in order to be compared on the same structural basis with the rest of the compositions. The ordered stoichiometric phases were calculated using PW and 4-atom supercells on an 18 × 18 × 18 Γ-centered *k*-point mesh to sample the Brillouin zone.^37^ All disordered Pt–Fe and Pt–Co phases were modeled in the *fcc* A1 phase, which was calculated using both PW and KKR-CPA. The PW calculations were based on large supercell models approximating the bulk disordered alloys, each of which was constructed using the SCRAPs method^22^ and consisted of 108 atoms. SCRAPs uses a hybrid cuckoo search algorithm that combinatorially optimizes atomic point and pair probabilities to generate a configuration with zero short-range order for up to a specified number (three in this study) of nearest-neighbor shells at every atomic site, with considerably higher computational efficiency than Monte Carlo-only methods. A 3 × 3 × 3 Γ-centered *k*-point mesh was used with the 108-atom SCRAPs supercells. For single-atom unit cells of monometallic Pt, Co, Ni, and Fe in the *fcc* structure, a 24 × 24 × 24 Γ-centered *k*-point mesh was used.

When KKR-CPA was used to calculate the disordered alloys, a single-atom primitive unit cell was sufficient for describing an *fcc* A1 phase. Two different MP *k*-point meshes were used with the KKR-CPA. For energy points with an imaginary part greater than 0.25 Ry, a 12 × 12 × 12 MP *k*-point mesh was used, and for energy points with an imaginary part less than 0.25 Ry (i.e., in the neighborhood of the Fermi level, ε_F_), an 18 × 18 × 18 MP *k*-point mesh was used.

Atomic H adsorption was modeled on *fcc*(111) slabs for all the metals and alloys considered in this study. Periodic slabs were separated by up to 14 Å of vacuum in the direction perpendicular to the surface. Adsorption at 1/4 ML was modeled with one H atom per (2 × 2) surface unit cell for the ordered phases. Adsorption at 1 ML was modeled with four H atoms per (2 × 2) surface unit cell for the ordered phases or 36 H atoms per (6 × 6) surface unit cell that was cut from the large SCRAPs supercell approximating one of the disordered alloys. The surface Brillouin zone was sampled on a 6 × 6 × 1 Γ-centered k-point mesh for the (2 × 2) surface unit cells or a 2 × 2 × 1 Γ-centered *k*-point mesh for the (6 × 6) surface unit cells. All surfaces were modeled as slabs containing four metal layers, with the bottom two layers kept fixed in their bulk positions and with the top two layers of metal atoms as well as all adsorbed H atoms fully relaxed until the maximum residual forces fell below 0.03 eV/Å in every relaxed degree of freedom. As all the metals and alloys studied herein are magnetic except monometallic Pt, care was taken that the total energy of each adsorption system was also minimized with respect to the spin degree of freedom by specifying different magnetic moments as initial guesses.

The average adsorption energy of atomic H was calculated as follows:
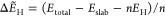
1where *E*_total_, *E*_slab_, and *E*_H_ are the total energies of the slab with adsorbed H, the clean slab, and a H atom in the gas phase, respectively, with *n* being the number of H atoms adsorbed in the surface unit cell. Gas-phase atomic H and molecular H_2_ were calculated in 10 × 10 × 10 Å^3^ simulation cells. The calculated H_2_ bond energy and bond length were 4.48 eV and 0.751 Å, respectively.

## 3 Results and Discussion

### 3.1 Bulk Lattice Constant and Energy

**Figure 1:**
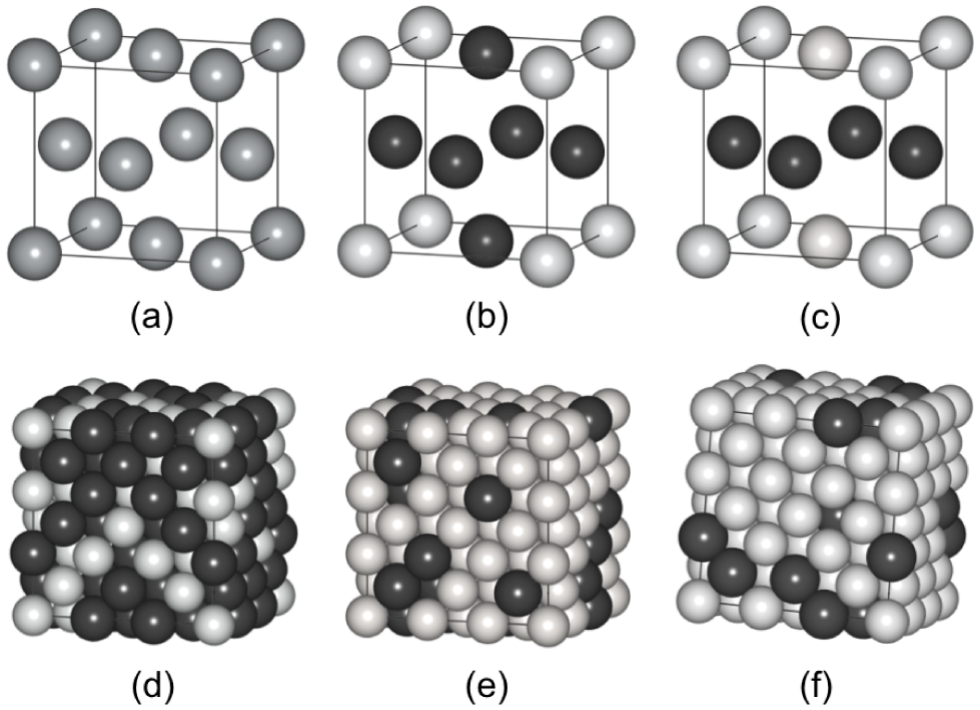
(a) *fcc* Structure for monometallic M (M = Fe or Co) and Pt; (b) L1_2_ structure for ordered PtM_3_ and Pt_3_M; (c) distorted L1_0_ structure for PtM with *c* = *a*. Large SCRAPs supercells in the *fcc* A1 structure consisting of (d) Pt_27_M_81_, (e) Pt_54_M_54_, and (f) Pt_92_M_16_. Color code: Pt = light spheres; M = dark spheres. Pt and Fe/Co in (d) and (f) are switched to obtain Pt_81_M_27_ and Pt_16_M_92_, respectively. Figure adapted with permission from ref.^21^, © 2020 Springer.

**Figure 2:**
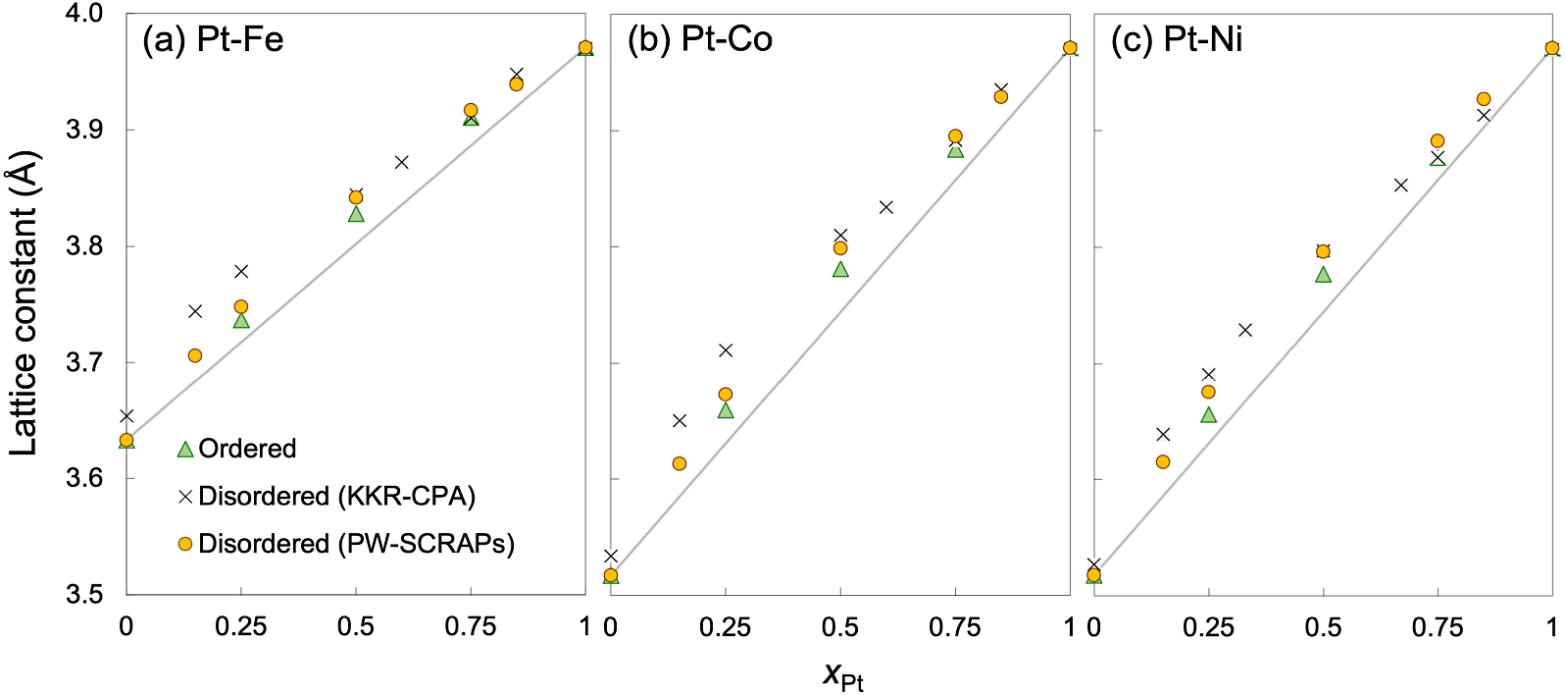
Calculated lattice constants of (a) Pt–Fe, (b) Pt–Co, and (c) Pt–Ni alloys plotted against *x*_Pt_. Lines connecting monometallic 3d metals and Pt represent Vegard’s law for respective alloys. Data for Pt–Ni taken in part from ref.^21^.

We begin by investigating certain bulk properties that are connected to catalytic properties, including the lattice constant and electronic structure. Figure 1 shows the models of the ordered and disordered Pt–Co and Pt–Fe alloy phases. The calculated equilibrium lattice constants are plotted in Figure 2 and listed in Table 1. The experimental values listed in Table 1 are taken from studies in which we are reasonably certain that a compositionally disordered A1 phase has been obtained, based on such considerations as annealing procedures and XRD results. Although GGA is known to overpredict lattice constants of metals, the theoretical results according to GGA-PBE are generally in close agreement with available experimentally measured lattice constants for both the ordered and disordered alloys. Due to the larger atomic radius of Pt, alloying it with the 3d base metals increases the lattice size compared to the monometallic 3d metals in the *fcc* phase, with higher Pt content (*x*_Pt_) resulting in a larger lattice constant. This is consistent with what we previously reported for Pt–Ni alloys.^21^

**Table 1:** Calculated Lattice Constants (in Å) of Ordered and Disordered Pt–Fe and Pt–Co Alloy Phases^a^

	*x*_Pt_	PW-ordered	PW-SCRAPs	KKR-CPA	ordered (exp.)	disordered (exp.)
Pt–Fe	0	3.633	-	3.654	3.639^39^	
	0.15	-	3.706	3.744	-	
	0.25	3.737	3.748	3.778	3.766,^40^ 3.727^38^	3.73^38^
	0.40	-			-	3.793^40^
	0.50	3.828	3.842	3.844		3.822,^40^ 3.84^38^
	0.60	-		3.872	-	3.854^40^
	0.75	3.911	3.917	3.910	3.874,^40^ 3.872^41^	3.88^38^
	0.85	-	3.939	3.948	-	
Pt–Co	0	3.517	-	3.534	3.537,^39^ 3.54^42^	
	0.15	-	3.613	3.650	-	
	0.25	3.660	3.673	3.711	3.664^43^	3.67^b^^18^
	0.50	3.781	3.799	3.810		3.782,^c^3.835^44^
	0.60	-		3.834	-	3.793,^d^^13^ 3.801,^e^^45^ 3.803^d^^1^
	0.75	3.884	3.895	3.892	3.831^39^	3.835,^f^^13^ 3.84^g^^18^
	0.85	-	3.929	3.935	-	
Pt	1	3.971		3.970	3.9233.920^46^^47^	

a Pure Fe, Co and Pt are calculated in the *fcc* structure. Ordered PtM_3_ and Pt_3_M are calculated in the L1_2_ structure. Ordered PtM is calculated in the distorted L1_0_ structure with *c* = *a*.

b Pt_0.24_Co_0.76_ reported in the A1 phase.

c Pt0.52Co0.48 reported in the A1 phase.

d Pt_0.63_Co_0.37_ reported in the A1 phase.

e Pt_0.58_Co_0.42_ reported in the A1 phase.

f Pt_0.73_Co_0.27_ reported in the A1 phase.

g Pt_0.77_Co_0.23_ reported in the A1 phase.

The lattice constants for the disordered Pt_0.25_Fe_0.75_, Pt_0.50_Fe_0.50_, and Pt_0.75_Fe_0.25_ alloys calculated with KKR-CPA are in close agreement with those reported by Vlaic and Burzo, who performed scalar relativistic tight-binding linear muffin-tin orbital (TB-LMTO) calculations in the local spin density approximation (LSDA) together with ASA and CPA. Their results were 3.718, 3.836, and 3.925 Å, respectively.^38^

The calculated lattice constants of both the ordered and disordered alloys show positive deviation from Vegard’s law, which means that a simple linear interpolation would underestimate the contribution by Pt–M interactions to the bulk volume of the Pt–M alloys, leading to underestimation of strain effects on surface reactivity. The predicted lattice constants can be fitted to a third-order polynomial for each alloy (Table 2). The nonlinear trends are in line with the lattice constants of disordered Pt–Ni and Pt–Co measured by Toda et al. as a function of atomic composition, while the lattice constants of Pt–Fe that they reported were much less sensitive to composition than our calculations indicate.^3^

**Table 2:** Calculated Lattice Constants for Disordered Pt–Fe, Pt–Co, and Pt–Ni Alloys Fitted to Third-Order Polynomials^a^

alloy	PW-SCRAPs	KKR-CPA
Pt–Fe	0.015*x*_Pt_^3^ – 0.184*x*_Pt_^2^ + 0.506*x*_Pt_ + 3.633	0.264*x*_Pt_^3^ – 0.537*x*_Pt_^2^ + 0.590*x*_Pt_ + 3.659
Pt–Co	0.038*x*_Pt_^3^ – 0.274*x*_Pt_^2^ + 0.690*x*_Pt_ + 3.517	0.367*x*_Pt_^3^ – 0.775*x*_Pt_^2^ + 0.846*x*_Pt_ + 3.537
Pt–Ni	0.089*x*_Pt_^3^ – 0.343*x*_Pt_^2^ + 0.707*x*_Pt_ + 3.518	0.371*x*_Pt_^3^ – 0.748*x*_Pt_^2^ + 0.819*x*_Pt_ + 3.528

a *R*^2^ > 0.99 in all cases.

The lattice constants of the disordered Pt_0.25_M_0.75_, Pt_0.50_M_0.50_, and Pt_0.75_M_0.25_ alloys calculated with PW-SCRAPs and KKR-CPA are larger than those of the ordered PtM_3_, PtM, and Pt_3_M alloys by up to 0.02 and 0.05 Å, respectively. It is typical of metallic alloys to increase in size when transitioning from an ordered to a disordered phase of the same composition.^34^ The lattice constants predicted by the PW calculations (ordered structures and SCRAPs) show the largest deviation from Vegard’s law around the midpoint in *x*_Pt_ (by up to 0.05 Å). The lattice constants for the disordered phases predicted by KKR-CPA, on the other hand, show the largest deviation from Vegard’s law at low *x*_Pt_ (by up to 0.08 Å). The KKR-CPA results are larger than the PW-SCRAPs results by the greatest amount: 1.0% for Fe and 0.7% for Ni at *x*_Pt_ = 0.15 and 1.0% for Co at *x*_Pt_ = 0.25.

The PW calculations show that the disordered A1 phases (based on the SCRAPs supercells) are consistently higher in energy than the corresponding ordered phases, which is in line with expectation. Johnson and coworkers^48,49^ have shown that the energy difference (δ*E*) between the ordered and disordered phases can be used to estimate the order/disorder transition critical temperature, *T*_C_, for a miscible alloy assuming a first-order transition, according to the following equation:
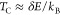
2

The estimated *T*_C_ agrees reasonably well with available experimentally measured *T*_C_ except for PtFe_3_ and Pt_3_Co (Table 3), for which *T*_C_ is severely underestimated. It suggests overstabilization of the disordered phase relative to the ordered phase, but the discrepancy has not been resolved despite our best effort.

**Table 3:** Difference in Total Energy between the Ordered and Disordered Phases (δ*E*, in meV/atom) and Estimated Order–Disorder Transition Critical Temperature (*T*_C_, in °C) for Pt–Fe and Pt–Co Alloys^a^

		*T*_C_	
composition	δ*E*	(est.)	(exp.)
PtFe_3_ → Pt_0.25_Fe_0.75_	66	488	835^50^
PtFe^b^ → Pt_0.50_Fe_0.50_	123	1154	1327,^48,50^ ∼1300^50^
Pt_3_Fe → Pt_0.75_Fe_0.25_	78	635	700–800^50^
PtCo_3_ → Pt_0.25_Co_0.75_	76	611	576^51^
PtCo^b^ → Pt_0.50_Co_0.50_	85	710	824,^45^ 825,^50,52^ 827^53^
Pt_3_Co → Pt_0.75_Co_0.25_	47	277	727,^53^ ∼750,^50^ 755^52^

a Results are based on the PW calculations.

b Ordered PtFe and PtCo are in the undistorted *fct* structure (*c* ≠ *a*).

### 3.2 Bulk Electronic Properties

**Figure 3:**
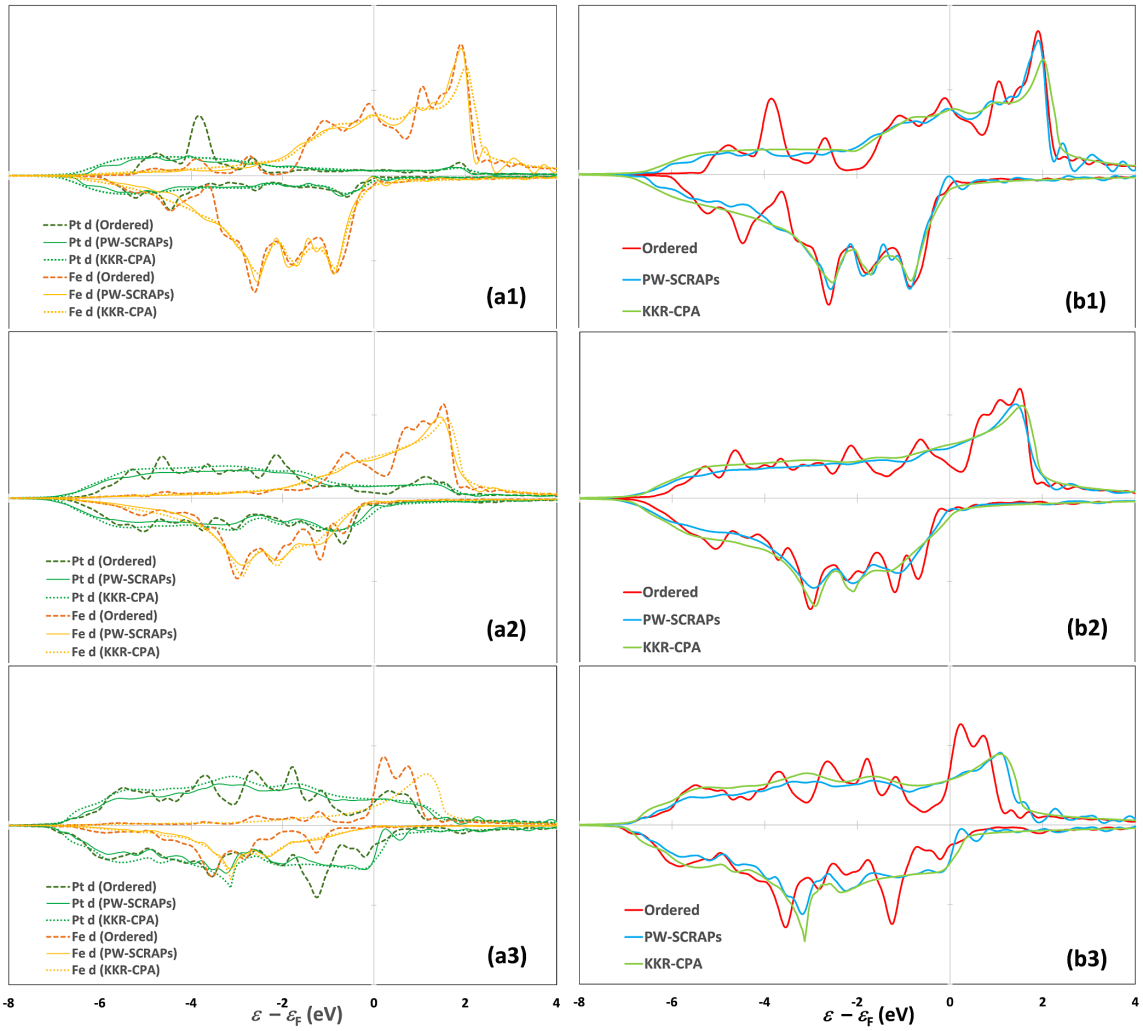
Project density of states (PDOS) for the Pt 5d and Fe 3d states normalized by the number of atoms of each element in a unit cell of (a1) ordered PtFe_3_ vs disordered Pt_0.25_Fe _0.75_; (a2) ordered PtFe vs disordered Pt_0.50_Fe_0.50_; (a3) ordered Pt_3_Fe vs disordered Pt_0.75_Fe _0.25_. (b1–b3) Total d PDOS normalized by the total number of atoms in a unit cell of the alloys in (a), calculated using different methods.

**Figure 4:**
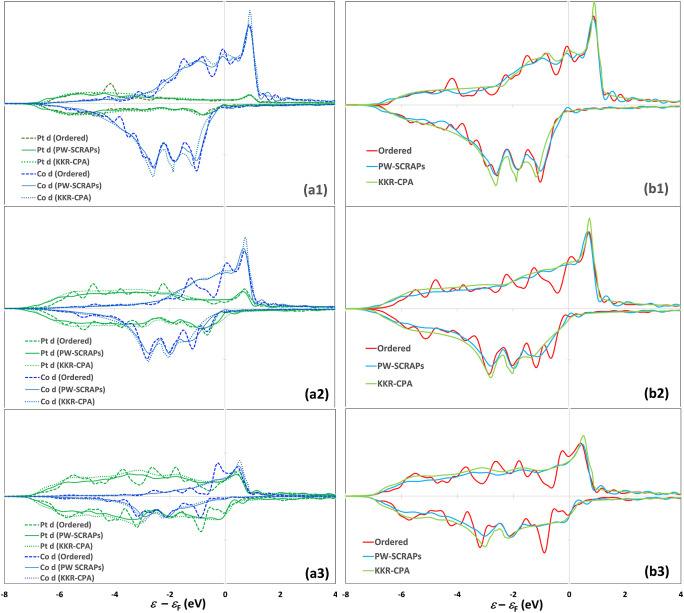
Project density of states (PDOS) for the Pt 5d and Co 3d states normalized by the number of atoms of each element in a unit cell of (a1) ordered PtCo_3_ vs disordered Pt_0.25_Co_0.75_; (a2) ordered PtCo vs disordered Pt_0.50_Co_0.50_; (a3) ordered Pt_3_Co vs disordered Pt_0.75_Co_0.25_. (b1–b3) Total d PDOS normalized by the total number of atoms in a unit cell of the alloys in (a), calculated using different methods.

In Figure 3, we plot and compare the total and element-specific d projected density of states (PDOS) for the ordered PtFe_3_, PtFe, and Pt_3_Fe and disordered alloys of the same composition based on SCRAPs and KKR-CPA approaches. The same is done for the Pt–Co alloys in Figure 4. Descriptors based on the bulk electronic structure are reported in Table 4, including the d band center (ε_d_) and magnetic moment. Note that these properties at surfaces may deviate from the bulk values listed here.

**Table 4:** Descriptors of Bulk Electronic Properties for Monometallic *fcc* Fe, Co, and Pt; Ordered PtFe_3_, PtFe, Pt_3_Fe, PtCo_3_, PtCo, and Pt_3_Co; and Disordered Pt_0.25_Fe_0.75_, Pt_0.50_Fe_0.50_, Pt_0.75_Fe_0.25_, Pt_0.25_Co_0.75_, Pt_0.5_Co_0.5_, and Pt_0.75_Co_0.25_: d Band Center (ε_d_, in eV, relative to ε_F_) and Average Magnetic Moment (in μ_B_/atom)^a^

	ordered		PW-SCRAPs		KKR-CPA		exp.
	ε_d_	mag. mom.	ε_d_	mag. mom.	ε_d_	mag. mom.	mag.mom.
Fe	+0.40	2.57					
Pt_0.15_Fe_0.85_	-	-	–0.18	2.32	–0.09	2.41	
			–2.97 (Pt)	0.36 (Pt)	–2.84 (Pt)	0.27 (Pt)	
			+0.26 (Fe)	2.66 (Fe)	+0.36 (Fe)	2.79 (Fe)	
PtFe_3_ or Pt_0.25_Fe_0.75_	–0.29	2.15	–0.51	2.13	–0.42	2.22	2.15^b^^54^
	–2.79 (Pt)	0.43 (Pt)	–2.93 (Pt)	0.35 (Pt)	–2.85 (Pt)	0.27 (Pt)	0.50^b^^54^
	+0.37 (Fe)	2.72 (Fe)	+0.22 (Fe)	2.72 (Fe)	+0.35 (Fe)	2.87 (Fe)	2.70^b^^54^
PtFe or Pt_0.50_Fe_0.50_	–1.05	1.66	–1.06	1.62	–1.14	1.68	
	–2.52 (Pt)	0.38 (Pt)	–2.54 (Pt)	0.33 (Pt)	–2.61 (Pt)	0.29 (Pt)	
	+0.22 (Fe)	2.94 (Fe)	+0.24 (Fe)	2.91 (Fe)	+0.25 (Fe)	3.07 (Fe)	
Pt_3_Fe or Pt_0.75_Fe_0.25_	–1.92	1.11	–1.77	1.02	–1.82	1.00	1.22^c^^55^
	–2.55 (Pt)	0.39 (Pt)	–2.48 (Pt)	0.32 (Pt)	–2.52 (Pt)	0.24 (Pt)	
	–0.33 (Fe)	3.26 (Fe)	+0.02 (Fe)	3.13 (Fe)	+0.10 (Fe)	3.28 (Fe)	3.3^b^^56^
Pt_0.85_Fe_0.15_	-	-	–2.21	0.68	–2.24	0.69	
			–2.62 (Pt)	0.25 (Pt)	–2.53 (Pt)	0.21 (Pt)	
			–0.05 (Fe)	3.21 (Fe)	–0.01 (Fe)	3.39 (Fe)	
Co	–0.82	1.67					
Pt_0.15_Co_0.85_	-	-	–1.13	1.50	–1.00	1.58	
			–3.37 (Pt)	0.36 (Pt)	–3.17 (Pt)	0.29 (Pt)	
			–0.78 (Co)	1.70 (Co)	–0.63 (Co)	1.80 (Co)	
PtCo_3_ or Pt_0.25_Co_0.75_	–1.28	1.45	–1.25	1.40	–1.18	1.48	
	–3.19 (Pt)	0.44 (Pt)	–3.16 (Pt)	0.36 (Pt)	–2.92 (Pt)	0.29 (Pt)	
	–0.72 (Co)	1.78 (Co)	–0.68 (Co)	1.75 (Co)	–0.60 (Co)	1.87 (Co)	
PtCo or Pt_0.50_Co_0.50_	–1.57	1.16	–1.68	1.13	–1.67	1.18	
	–2.56 (Pt)	0.43 (Pt)	–2.75 (Pt)	0.36 (Pt)	–2.68 (Pt)	0.32 (Pt)	
	–0.57 (Co)	1.89 (Co)	–0.61 (Co)	1.89 (Co)	–0.58 (Co)	2.03 (Co)	
Pt_3_Co or Pt_0.75_Co_0.25_	–2.06	0.75	–2.14	0.74	–2.13	0.77	0.61^b^^57^
	–2.57 (Pt)	0.35 (Pt)	–2.56 (Pt)	0.31 (Pt)	–2.57 (Pt)	0.29 (Pt)	0.26^b^^57^
	–0.63 (Co)	1.97 (Co)	–0.60 (Co)	2.03 (Co)	–0.62 (Co)	2.18 (Co)	1.64^b^^57^
Pt_0.85_Co_0.15_	-	-	–2.31	0.56	–2.12	0.57	
			–2.65 (Pt)	0.29 (Pt)	–2.57 (Pt)	0.27 (Pt)	
			–0.65 (Co)	2.10 (Co)	–0.66 (Co)	2.26 (Co)	
Pt	–2.71	-					

a The ε_d_ and magnetic moment not designated with an element are overall values for a metal/alloy. For calculation of ε_d_, the d band is integrated from the lowest energy up to where the PDOS (occupied or unoccupied) drops below 5% of its maximum value for the first time. The ε_d_ of the spin-up channel is reported here.

b Ordered phases.

c Disordered phase with *x*_Pt_ = 0.737.

Both PW-SCRAPs and KKR-CPA predict all the alloy compositions to be ferromagnetic instead of nonmagnetic. The average total and element-specific magnetic moments for the ordered and disordered phases of the same compositions are similar, and they are in line with available experimental measurements (Table 4)^54−56,58,59^ and theoretical work on ordered Pt–Fe and Pt–Co alloys.^38,43,60,61^ In agreement with previous studies,^55^ the average bulk magnetic moment of the Pt–Fe and Pt–Co alloys decreases with increasing *x*_Pt_, but these alloys remain ferromagnetic even at high *x*_Pt_. The same trends and similar magnetic moments have been obtained by Vlaic and Burzo by performing TB-LMTO-CPA calculations for disordered Pt–Fe A1 phases.^38^ It should be noted that for each disordered alloy, KKR-CPA yields a configurational average not only in the atomic composition but also in the magnetic composition, while PW-SCRAPs cannot do so effectively.

The d PDOS of the ordered alloys exhibits sharp features, while that of the disordered alloys is smooth and shows few features. Yet, many similarities are seen in how the total PDOS and the element-specific d PDOS are distributed in the ordered vs disordered Pt–Fe and Pt–Co alloys. The 3d states of Fe and Co are distributed across the ε_F_ between ca. −2 eV and +2 eV in the spin-up channel but are concentrated below the ε_F_ down to ca. −4 eV in the spin-down channel. The 5d band of Pt exhibits notable density of states above ε_F_ due to hybridization with the 3d states of Fe and Co in the spin-up channel causing the Pt ε_d_ to exceed that of monometallic Pt at *x*_Pt_ > 0.5, while it is spread out evenly between the ε_F_ and −7 eV in the spin-down channel. This is also reflected in the appreciable magnetic moment on Pt that is nearly invariant with composition. The center (ε_d_) of the total spin-up d band varies continuously between the monometallic limits for both the disordered and ordered phases. The PW calculations based on the large SCRAPs supercells agree closely with KKR-CPA on the disordered phases.

As *x*_Pt_ increases, the split between the spin-up and spin-down 3d bands widens, and the magnetic moments of the 3d metal atoms increase significantly beyond those of the bulk base metals. These characteristics, plus the fact that the 3d metal atoms are all smaller than Pt, suggest a quasi-free atom-like interpretation^62^ of the 3d metal atoms in the Pt-rich limit. If we focus on ε_d_ of Pt at high *x*_Pt_, a trend can be identified across the Pt–Fe, Pt–Co, and Pt–Ni alloys. At *x*_Pt_ = 0.85, the ε_d_ of the 5d band of Pt (PW-SCRAPs) is −2.62 eV for Pt–Fe, −2.65 eV for Pt–Co, and −2.75 eV for Pt–Ni, the last of which being the closest to monometallic Pt. This is so even though Pt–Fe experiences the least amount of compressive strain for a given *x*_Pt_ among Pt–Fe, Pt–Co, and Pt–Ni (cf. Figure 1), suggesting that the electronic perturbation by Fe on Pt is the greatest among the three groups of alloys. Consistent with this interpretation, the leading edge of the Fe spin-up 3d band falls back toward ε_F_ with increasing *x*_Pt_, while that of Co retracts only slightly. Consequently, the ε_d_ of the Co 3d band varies mildly with *x*_Pt_, but the ε_d_ of the Fe 3d band decreases notably with increasing *x*_Pt_.

Toward the other limit, at *x*_Pt_ = 0.15, ε_d_ of the 3d band of Fe is +0.26 eV (vs +0.40 eV for bulk Fe); that of Co is −0.78 eV (vs −0.82 eV for bulk Co); and that of Ni is −1.13 eV (vs −1.29 eV for bulk Ni). The largest upshift is seen for Ni. This is taken to be the combined effects of expansive strain (which is most significant in Pt–Ni) and, consistent with the above, weaker interactions between Pt–Ni than Pt–Fe and Pt–Co. At the same time, narrowing of the Pt 5d band is not seen with decreasing *x*_Pt_. This is because when the base metal concentration is enriched and the lattice contracts, Pt atoms are compressed into stronger interaction with the 3d metal atoms.

### 3.3 Hydrogen Adsorption on the (111) Facets of Metals and Alloys

**Figure 5:**
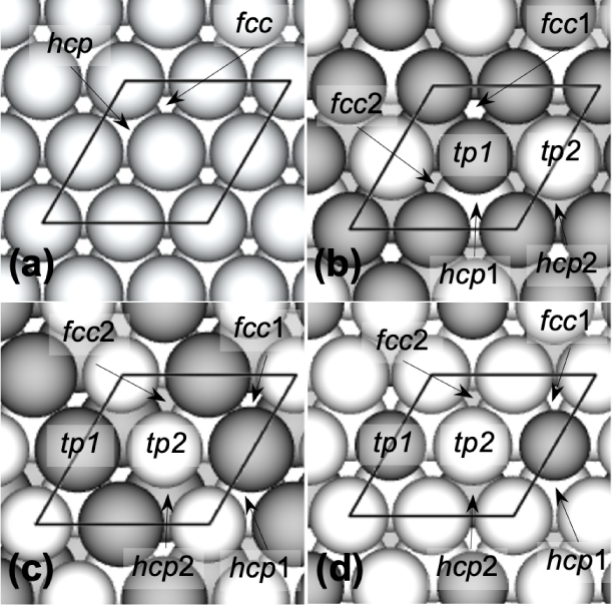
Top views of the (111) surface models for (a) monometallic Pt and M (M = Fe or Co), (b) ordered PtM_3_, (c) ordered PtM, and (d) ordered Pt_3_M. Color code: Pt = light spheres; M = dark spheres. High-symmetry adsorption sites considered in this study are labeled. The (2 × 2) surface unit cells are outlined. Figure adapted with permission from ref.^21^, © 2020 Springer.

The adsorption energy of atomic H (Δ*E*_H_) on the (111) facets of monometallic Co, Fe, and Pt and of the ordered Pt–Fe and Pt–Co alloys is reported in Table 5. The surface models for the ordered Pt–Fe and Pt–Co alloys are illustrated in Figure 5. There are two different *fcc* 3-fold hollow sites and two different *hcp* 3-fold hollow sites on the (111) facet of each ordered Pt–M alloy, based on the composition of the three metal atoms that make up each site. For each type of *fcc* site, a site with more base metal atoms (Fe or Co) is designated as “1” and the other is designated as “2”. Thus, on a PtM_3_(111) surface, *fcc*1 and *fcc*2 stand for M_3_ and M_2_Pt sites, respectively; on a PtM(111) surface, they stand for M_2_Pt and MPt_2_ sites, respectively; and on a Pt_3_M(111) surface, they stand for MPt_2_ and Pt_3_ sites, respectively. At one-fourth ML coverage, the *fcc*1 site is the most stable adsorption site for atomic H on all the ordered alloy (111) facets investigated in this study, in all cases followed by the *hcp*2 site except PtFe_3_.

**Table 5:** Adsorption Energy of Atomic H (Δ*E*_H_ at 1/4 ML and  at 1 ML; Both in eV) on the (111) Facets of Monometallic *fcc* Fe, Co, and Pt and of Ordered Pt–Fe and Pt–Co Alloy Phases at Respective Equilibrium Lattice Constants, in Comparison with Literature Values^a^

		Fe	PtFe_3_	PtFe	Pt_3_Fe	Co	PtCo_3_	PtCo	Pt_3_Co	Pt
1/4 ML	*fcc*/*fcc*1	–2.99	–2.91	–2.76	–2.71	–2.79	–2.79	–2.71	–2.68	–2.73
	*fcc*2		–2.80	–2.65	–2.60		–2.69	–2.62	–2.60	
	*hcp*/*hcp*1	–2.98	–2.85	–2.66	–2.60	–2.77	–2.67	–2.62	–2.61	–2.69
	*hcp*2		–2.78	–2.68	–2.66		–2.69	–2.63	–2.62	
	*top*/*top*1	–2.60	(*fcc*1)	(*fcc*1)	–1.85	–2.13	(*fcc*1)	(*hcp*2)	–2.05	–2.70
	*top*2		–2.57	(*fcc*1)	–2.61		–2.46	(*fcc*1)	–2.62	
1 ML	*fcc*	–2.96	–2.76	–2.62	–2.55	–2.73	–2.64	–2.60	–2.60	–2.65
	*top*	–1.92	–1.98	(*fcc*)	–2.33	–1.93	–2.61	–2.19	–2.38	–2.61
PBE										*–0.50*(63)–2.88^64^
PW91		–2.99,^b^^65^–3.02^b^^66^			–*0.45*^67^	–2.89^c^^65^			–2.67^68^	–2.72,^65^–2.74^69^
RPBE		–2.78,^b^^65^–2.86^b^^66^				–2.69^c^^65^				–2.55,^65^–2.57^69^
exp.		–*0.69*,^70^–2.59^70^								–*0.80*,^70^–*0.44*,^70^–2.78^71^

a ΔẼ_H_ is the average of all fcc sites. Values in italics are referenced to molecular H_2_ instead of atomic H. The literature DFT values are those for atomic H in the *fcc* site and are grouped by the type of GGA functional used.

b bcc Fe.

c hcp Co.

**Figure 6:**
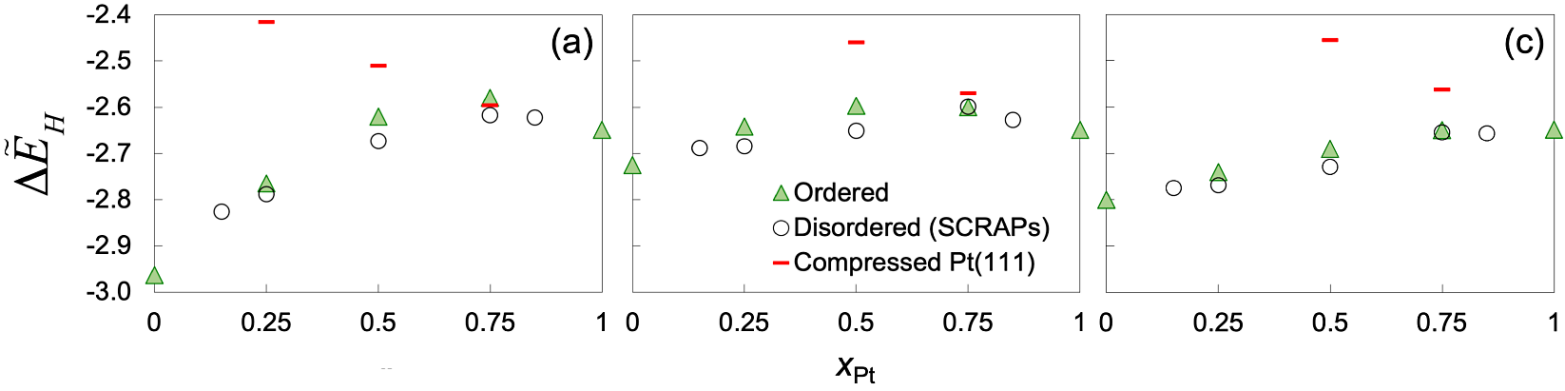
Average adsorption energy () for 1 ML of atomic H on all *fcc* sites of the (111) facets of ordered and disordered (a) Pt–Fe, (b) Pt–Co, and (c) Pt–Ni alloys plotted against the atomic concentration of Pt (*x*_Pt_). Δ*E*_H_ on strained monometallic Pt(111) at the same lattice constants as those of the ordered phases is included for comparison. All results here are obtained using PW-DFT.

The average adsorption energy () for 1 ML of atomic H on all *fcc* sites of the ordered (111) facets is plotted in Figure 6 (filled triangles). Results at 1 ML are reported because in many hydrogen-based catalytic applications, sufficiently reactive surfaces such as these alloys readily acquire high surface coverages of H in a catalytically active state.  generally becomes less negative with increasing *x*_Pt_. It varies roughly linearly with composition for Pt–Ni but is clearly not linear for Pt–Fe and Pt–Co, where a maximum occurs around *x* = 0.5–0.75. This is so despite the fact that monometallic Fe adsorbs H notably more strongly than Ni.

Figure 6 appears to suggest that the direct contribution of the base metal to adsorption wanes with diminishing base metal concentration, but an indirect contribution persists through a contracted lattice such that at large *x*_Pt_, Pt–Fe and Pt–Co surfaces behave like compressed Pt surfaces (Figure 6, red bars). Xu et al. previously attributed the fact that the adsorption of atomic O on the *fcc*2 site (all Pt) on Pt_3_Fe and Pt_3_Co is weaker than that on monometallic Pt, to compressive strain.^72^ However, as seen in Table 5, there is a ca. 0.1 eV difference in Δ*E*_H_ between different *fcc* sites on Pt_3_Fe and Pt_3_Co (0.05 eV on Pt_3_Ni).^21^ As will be discussed below, the distribution of site reactivity is more complex than  suggests.

**Figure 7:**
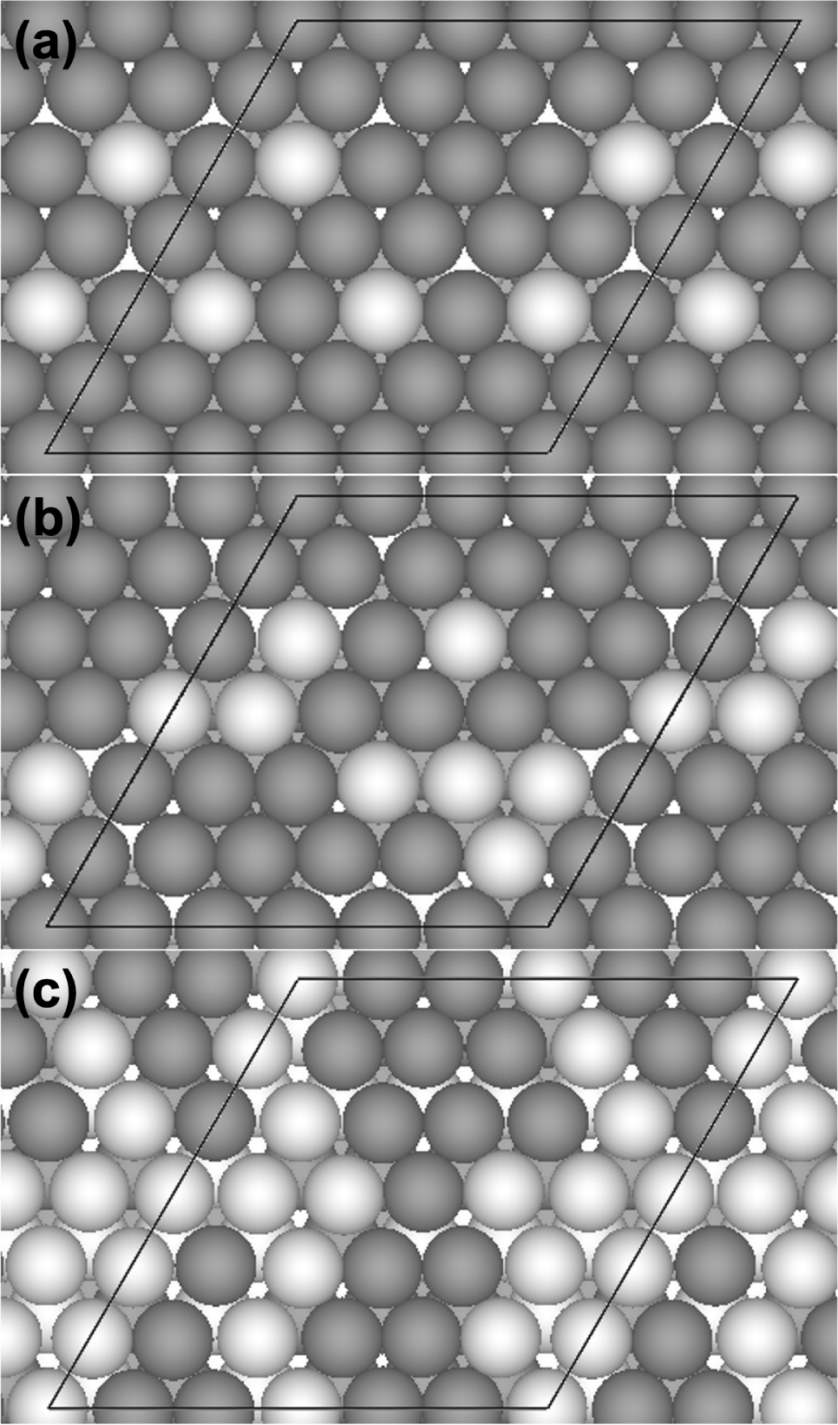
Top views of the (6 × 6) (111) slabs of (a) Pt_0.15_M_0.85_, (b) Pt_0.25_M_0.75_, and (c) Pt_0.50_M_0.50_. The surface unit cells are outlined. Color code: Pt = light spheres; M = dark spheres. Pt and M in (a, b) are switched to obtain the corresponding (111) slabs of Pt_0.85_M_0.15_ and Pt_0.75_M_0.25_, respectively. The numbers of surface Pt atoms in the (6 × 6) unit cells is (a) 5, (b) 8, and (c) 17.

The large (6 × 6) surface unit cells that are cut from the respective SCRAPs supercells to represent the (111) facets of the disordered phases are illustrated in Figure 7. Each surface model is constructed at the respective equilibrium lattice constant as determined in Section 3.1. The compositional randomness of the disordered alloys means that many more inequivalent *fcc* sites exist than on the surfaces of the ordered alloy phases. Based solely on the composition of the first coordination shell, four different *fcc* sites may be found on a (111) binary alloy surface, i.e., M_3_, Pt_1_M_2_, Pt_2_M_1_, and Pt_3_. In the limit of complete randomness, the statistical distribution of these four *fcc* sites is a function of the bulk composition.^73,74^ Each slab is cut so that the exposed top surface expresses a distribution of the different types of *fcc* sites that is as close to the limiting value (Table 6) as possible, with an atomic composition that is as close to the bulk composition as possible. Note that these are not typical *fcc*(111) slabs in the sense that, while the stacking follows an ABCA··· pattern, the composition per layer does not repeat as such.

**Table 6:** Distributions of Different Types of *fcc* 3-fold Site (Based on the Composition of the First Coordination Shell) on the (111) Facet of Random Pt–M alloys as a Function of Bulk composition, Given as Relative Concentrations of M_3_: M_2_Pt: MPt_2_: Pt_3_

	ideal limit^a^	(6 × 6) slabs^b^
Pt_0.15_M_0.85_	0.61:0.33:0.06:0.00	0.58:0.42:0:0
Pt_0.25_M_0.75_	0.42:0.42:0.14:0.02	0.47:0.42:0.08:0.03
Pt_0.50_M_0.50_	0.13:0.38:0.38:0.13	0.19:0.33:0.33:0.14

a Refs^73,74^.

b This study.

 of 1 ML of atomic H adsorbed on all *fcc* sites on each of the disordered alloy surfaces is calculated and compared with the ordered surfaces in Figure 6 (open circles). Snapshots of the minimum-energy adsorption structures of 1 ML of H atoms on several Pt–Fe (111) surfaces are illustrated Figure S1. In terms of , the disordered alloy surfaces adsorb H somewhat more strongly than the ordered alloy surfaces of the same compositions, but overall, the two sets of surfaces exhibit similar dependence on *x*_Pt_ for each base metal. ft As on the ordered alloy surfaces, weaker  than monometallic Pt occurs at intermediate *x*_Pt_ values, such that a maximum forms at *x*_Pt_ = 0.75 with Pt–Fe and Pt–Co. The same is not seen for Pt–Ni.

To probe the variation in individual site reactivity on the disordered surfaces, we sample the differential adsorption energy for atomic H on individual sites at 1 ML coverage, defined as follows:
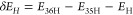
3

**Figure 8:**
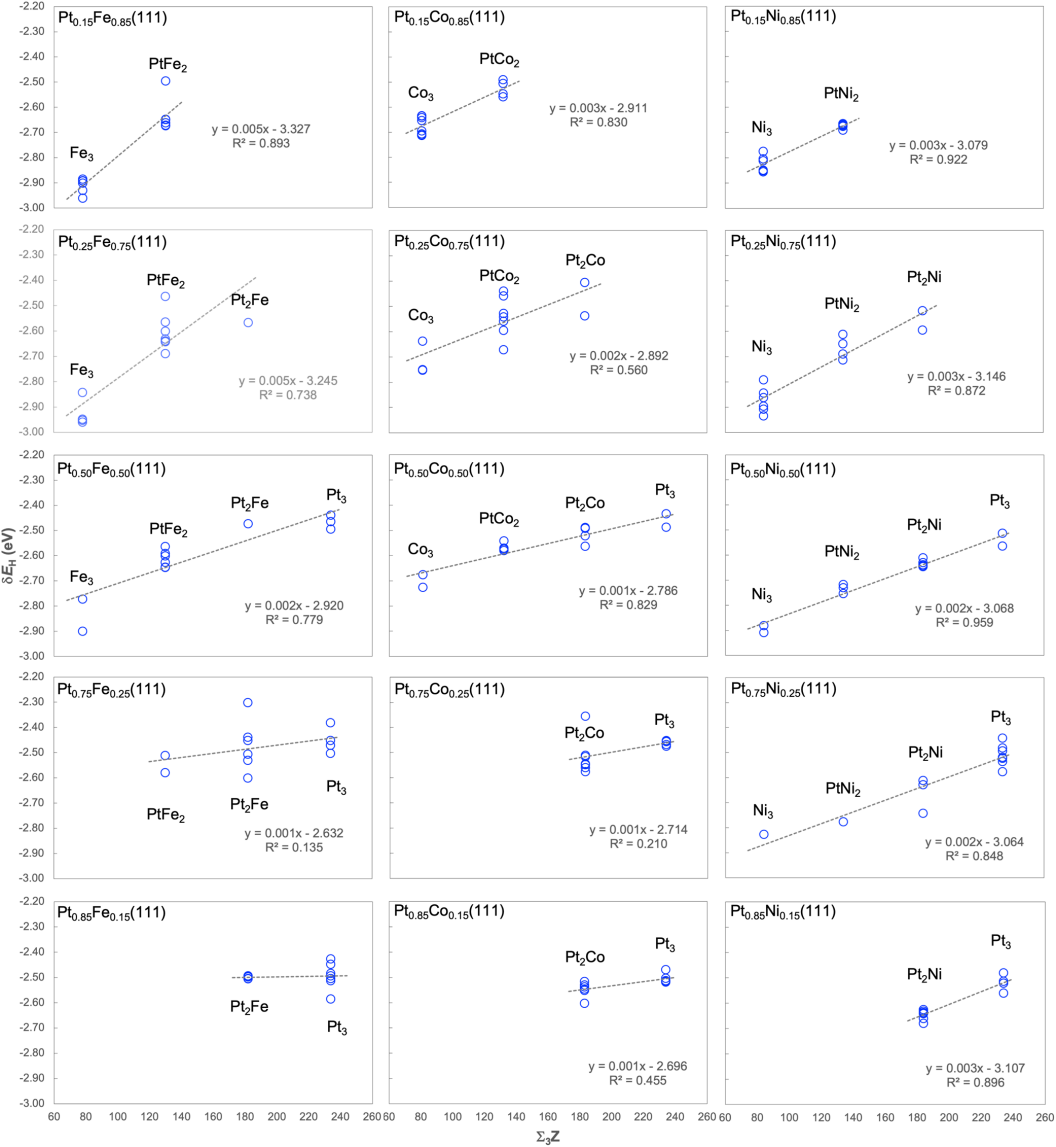
Differential adsorption energy of atomic H (δ*E*_H_) at 1 ML coverage on the (111) facets of disordered (left) Pt–Fe, (center) Pt–Co, and (right) Pt–Ni alloys plotted against the sum of atomic numbers of the three metal atoms comprising each site (Σ_3_Z). *x*_Pt_ increases from top to bottom panels. Results are based on a randomly chosen subset of one-third of the *fcc* sites on each surface.

In our recent study involving oxygen adsorption on a high-entropy alloy surface, CoCrFeNi(111), the sum of the atomic numbers of the metal atoms that make up an *fcc* 3-fold site (∑_3_Z) was found to be a good descriptor for the differential adsorption energy for atomic O.^75^ Here, we plot δ*E*_H_ against ∑_3_Z in Figure 8. Since there are two metal elements in each group of alloys, there are only up to four discrete values that correspond to, in an increasing order of ∑_3_Z, M_3_, M_2_Pt, MPt_2_, and Pt_3_ sites.

An approximately linear correlation appears between these two quantities on all three groups of disordered Pt alloys. One trend that emerges is that the slope of the linear correlations generally decreases with increasing *x*_Pt_, which is most obvious for Pt–Fe and least so for Pt–Ni. A spread of 0.1–0.3 eV is seen for most site types, which suggests that the effects due to factors such as size, electronegativity, and detailed electronic structure of the metal atoms on δ*E*_H_ are perturbative and limited to this extent. There are a handful of outlying data points that clearly do not conform to the correlations, which have been confirmed after close inspection. H adsorption energy on metal oxides has been found to be correlated with the Bader charge of the O sites that they occupy.^76^ In the present study, there is a nearly perfectly linear correlation between the sum of Bader charges and the ∑_3_Z of the metal atoms comprising the *fcc* sites on each disordered alloy surface considered (*R*^2^ > 0.99; not shown), so the Bader charge of the sites would not be a better descriptor for δ*E*_H_ than ∑_3_Z.

**Figure 9:**
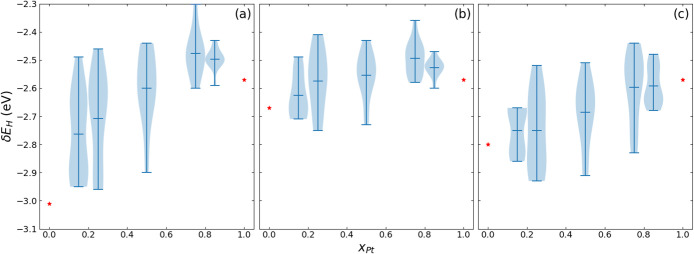
Violin plots of differential adsorption energy of atomic H (δ*E*_H_) at 1 ML coverage on the (111) facets of (a) Pt–Fe, (b) Pt–Co, and (c) Pt–Ni random alloys from Figure 8 vs the atomic concentration of Pt (*x*_Pt_). Red stars indicate δ*E*_H_ at 1 ML on the monometallic surfaces. The mean in each distribution is indicated.

The results shown in each panel of Figure 8 are combined into one violin plot to illustrate the overall distributions of δ*E*_H_ and heterogeneity in surface reactivity as a function of alloy composition for Pt–Fe (Figure 9a) as well as for Pt–Co and Pt–Ni (Figure 9b,c). The spread in δ*E*_H_ is the smallest at the Pt-rich limit and is larger at the intermediate *x*_Pt_ values for all three 3d metals. The Pt–Fe surfaces differ from Pt–Co and Pt–Ni in that the spread in δ*E*_H_ does not decrease with decreasing *x*_Pt_. Overall, the difference in the limiting δ*E*_H_ values being the smallest for monometallic Co vs Pt corresponds to the smallest spreads in δ*E*_H_ on the Pt–Co surfaces, while the opposite is true for Pt–Fe.

For each surface, the base metal-rich sites are located toward the bottom of each distribution, while the Pt-rich sites are located toward the top. For all three base meals, as *x*_Pt_ decreases from 1, the top end of the distribution rises considerably above the monometallic Pt limit. This is particularly so for Pt–Fe and Pt–Co (Figure 9a,b), while the distributions on the Pt_0.85_Ni_0.15_ and Pt_0.75_Ni_0.25_ surfaces (Figure 9c) are lower than those on the corresponding Pt–Fe and Pt–Co surfaces. Thus, a maximum forms in mean δ*E*_H_ vs *x*_Pt_ for Pt–Fe and Pt–Co but not for Pt–Ni, similar to  (Figure 6). Conversely, as *x*_Pt_ increases from 0, the bottoms of the distributions sink below the monometallic Co and Ni limits, while the latter effect is not visible for Pt–Fe.

Based on lattice constants (Figure 1), Pt-rich sites experience increasing compressive strains as *x*_Pt_ decreases from 1, while the base metal-rich sites are subject to expansive strains as *x*_Pt_ increases from 0. Because of the smaller difference in lattice constant between Fe/Pt than between Co/Pt and Ni/Pt, one might expect Pt-rich sites to be subject to less compressive strain on the disordered Pt–Fe surfaces than on the disordered Pt–Ni surfaces. However, while Pt-rich sites become less reactive toward H with decreasing *x*_Pt_ on all three groups of disordered Pt–M alloy surfaces, those on Pt–Fe are the least reactive.

We attribute this phenomenon to the ligand effect, i.e., stronger interaction between Fe/Pt than between Co/Pt followed by Ni/Pt,^77^ which reduces the reactivity of both the base metal and Pt, more so for Fe/Pt than for Ni/Pt. This interaction is reflected in notably higher *T*_C_ for Pt–Fe and Pt–Co than Pt–Ni for medium to high *x*_Pt_. It contributes a further passivating effect to the Pt-rich sites, while also causing the Fe-rich sites on the Pt–Fe surfaces to be less reactive relative to Fe than the Ni-rich sites on the Pt–Ni surfaces are relative to Ni.

For Pt–Fe and Pt–Co, due to the existence of a maximum in  and δ*E*_H_ vs *x*_Pt_, the average adsorption strength of H is degenerate in a certain range of *x*_Pt_ with two possible materials solutions, one being Pt-lean and the other being Pt-rich. If one desires to achieve a certain  in, e.g., −2.62 to −2.67 eV (cf. Figure 6) using a random Pt–Fe alloy, a Pt-rich solution with *x*_Pt_ > 0.75 would be preferable in the sense that it offers a narrower distribution in site reactivity than the other solution with *x*_Pt_ < 0.75.

### 3.4 Bader Charge of Adsorbed Hydrogen

**Figure 10:**
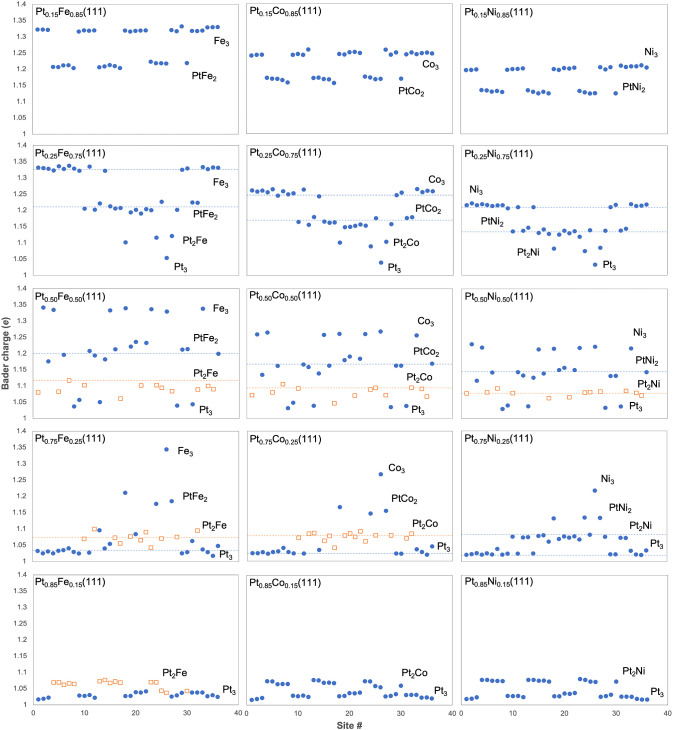
Bader charges of 36 individual H atoms adsorbed at 1 ML coverage on all *fcc* sites of the (111) facets of disordered (left) Pt–Fe, (center) Pt–Co, and (right) Pt–Ni alloys. *x*_Pt_ increases from top to bottom panels. The neutral, free H atom has a charge of 1 *e*. Site types are as labeled. In some of the panels, data points representing Pt_2_M sites are represented by a different color and symbol to distinguish them from the other site types. Horizontal dashed lines represent average Bader charges of H atoms adsorbed on the indicated site types on ordered Pt–M alloys of the same compositions.

Bader charge analysis provides additional insights into H adsorption on the disordered alloy surfaces. The results for the Pt–Fe surfaces are shown in Figure 10. Overall, the trends in BC_H_ and δ*E*_H_ parallel each other in some ways. The spread in BC_H_ is smaller at the monometallic limits (Pt_0.15_M_0.85_ and Pt_0.85_M_0.15_, except for Pt_0.15_Fe_0.85_) and is larger at intermediate *x*_Pt_, just like δ*E*_H_.

All H atoms are seen as negatively charged, i.e., hydride species. In most cases, distinct levels of BC_H_ are seen that are readily identified with the different site types, which more clearly reveals than what the underlying electronic structure indicates, different H–metal interaction states (Figure S2). For instance, BC_H_ separates into two levels for Pt_0.15_Fe_0.85_(111): the majority level (ca. 1.32) occurs on all-Fe (Fe_3_) sites, while the minority level (ca. 1.2) corresponds to all the PtFe_2_ sites. Because Fe is less electronegative than Pt, H atoms residing on Fe_3_ sites acquire more charges than those in the PtFe_2_ sites. For Pt_0.25_Fe_0.75_(111), four levels of BC_H_ are identifiable. Fe_3_ and PtFe_2_ are the most and second most numerous and most negatively charged sites for H, followed by Pt_2_Fe and finally all-Pt sites (Pt_3_). Variation in BC_H_ within a site type intensifies with increasing *x*_Pt_ because sites with identical composition in the first coordination shell can have different compositions in outer shells. Moreover, H atoms are in close proximity to the metal atoms beneath the *fcc* sites, and variation in the subsurface composition also increases as the composition moves away from the dilute Pt limit. Overall, the variation in BC_H_ reaches its greatest extent at *x*_Pt_ = 0.5 and then narrows again. This pattern qualitatively parallels the distribution in δ*E*_H_ on the disordered Pt–Fe (111) surfaces (Figure 9a). When *x*_Pt_ exceeds 0.5, nevertheless, the pattern reverses but is not the mirror opposite of Pt_0.15_Fe_0.85_ and Pt_0.25_Fe_0.75_. For *x*_Pt_ ≥ 0.5, BC_H_ values for the Pt_2_Fe and Pt_3_ sites are not distinct from each other.

BC_H_ on the Pt–Co and Pt–Ni surfaces exhibits similar patterns, the main difference being that the overall spread in BC_H_ across the different site types for *x*_Pt_ ≤ 0.75 is progressively smaller on the Pt–Co and Pt–Ni surfaces than on the corresponding Pt–Fe surfaces. This is consistent with the fact that the electronegativity of the 3d transition metals increases as one moves toward the right of the periodic table, narrowing the difference vs Pt. There is no consistent numerical correspondence between the spread in BC_H_ and the spread in δ*E*_H_.

## 4 Conclusions

To explore Pt–Fe and Pt–Co bimetallic alloys as potential compositionally tunable catalysts, we have theoretically investigated several *fcc* Pt–Fe and Pt–Co alloy phases, including ordered PtM_3_, PtM, and Pt_3_M and disordered Pt_0.15_M_0.85_, Pt_0.25_M_0.75_, Pt_0.50_M_0.50_, Pt_0.75_M_0.25_, and Pt_0.85_M_0.15_, using a combination of PW-DFT and KKR-CPA. Bulk properties and surface reactivity in terms of atomic H adsorption were calculated. PW-DFT calculations of the disordered phases were based on large structural models generated using the SCRAPs algorithm. The bulk lattice constant appears to be a smooth function of atomic composition that clearly deviates from Vegard’s law for both Pt–Fe and Pt–Co, just as Pt–Ni. The deviation is more pronounced in a mid-to-low *x*_Pt_ regime, and the disordered phases have larger lattice constants than the order phase of the same composition. Our results in the dilute regimes, i.e., Pt_0.15_M_0.85_ and Pt_0.85_M_0.15_, may also be pertinent to research involving single-atom alloys (SAAs) of these Pt–M combinations.

While the total d band center, ε_d_, varies monotonically between the pure metal limits for both the disordered and ordered phases for each Pt–M combination, intricate changes are revealed when the electronic structure is decomposed by elements. We present evidence that factors including disparity in the atomic radii, lattice strain, spin polarization, and compositional order/disorder all affect how the d band of each element evolves with composition. Pt is hybridized with the base metals over the entire composition range, with the ε_d_ of the Pt 5d band rising above that of the pure Pt level at mid-to-high *x*_Pt_ due to significant density of states that the base metals have at and above the Fermi level. This also causes even the Pt-rich Pt_3_M phases to be mildly ferromagnetic.

In terms of the average adsorption energy of atomic H, , the ordered and disordered surfaces of the same composition appear similar in chemical reactivity. The disordered surfaces fill in the trends outlined by the ordered surfaces, which suggests that  can be continuously tuned to any desired value between the monometallic limits for each pair of Pt–M. The reactivity of individual *fcc* adsorption site toward H (as represented by the differential adsorption energy, δ*E*_H_) is found to be closely related to the sum of the atomic numbers of the metal atoms that comprise a site (∑_3_Z), and the dependence weakens with increasing *x*_Pt_ for a given base metal. The difference in surface reactivity is the smallest between monometallic Co and Pt; the smallest overall distribution in site reactivity is seen on the Pt–Co surfaces, while the opposite is true for Pt–Fe. Analysis of the distribution of δ*E*_H_ suggests that strong base metal–Pt electronic interactions supplant compressive lattice strains to reduce surface reactivity, so that a maximum forms in average Δ*E*_H_ and δ*E*_H_ with respect to *x*_Pt_ for Pt–Fe and Pt–Co, but not for Pt–Ni. Consequently, degenerate material solutions may exist for average adsorption strength of H in a certain range of *x*_Pt_, with *x*_Pt_ > 0.5 offering tunability in  with narrower distributions. The results here also provide a context for understanding the interaction of other adsorbates with Pt–M alloys and shed light on the surface reactivity of more complex, higher-order random alloys and how it should be conceptualized and harnessed for catalytic applications.^75,78−80^

## Supporting Information Available

Structure and PDOS of DFT-optimized 1 ML of H atoms adsorbed on several disordered Pt-M surfaces (PDF)
