# Supplementary material for: Hydrogen Adsorption on Ordered and Disordered Pt–Fe and Pt–Co Alloys
Source: J Phys Chem C Nanomater Interfaces. 2024 Jun 29;128(27):11145–58. doi: 10.1021/acs.jpcc.4c01308 (PMC11247490; doi:10.1021/acs.jpcc.4c01308)
Supplement: Supplementary file 1 — jp4c01308_si_001.pdf [file jp4c01308_si_001.pdf]

Hydrogen Adsorption on Ordered and Disordered Pt-Fe and Pt-Co Alloys

Andrew Okafor,[1] William A. Shelton,[2] and Ye Xu[*1]

[1]Cain Department of Chemical Engineering, and [2]Department of Physics and Astronomy,

Louisiana State University, Baton Rouge, LA 70803, USA.

Supporting Information

[*] Corresponding author. E-mail: yexu@lsu.edu.

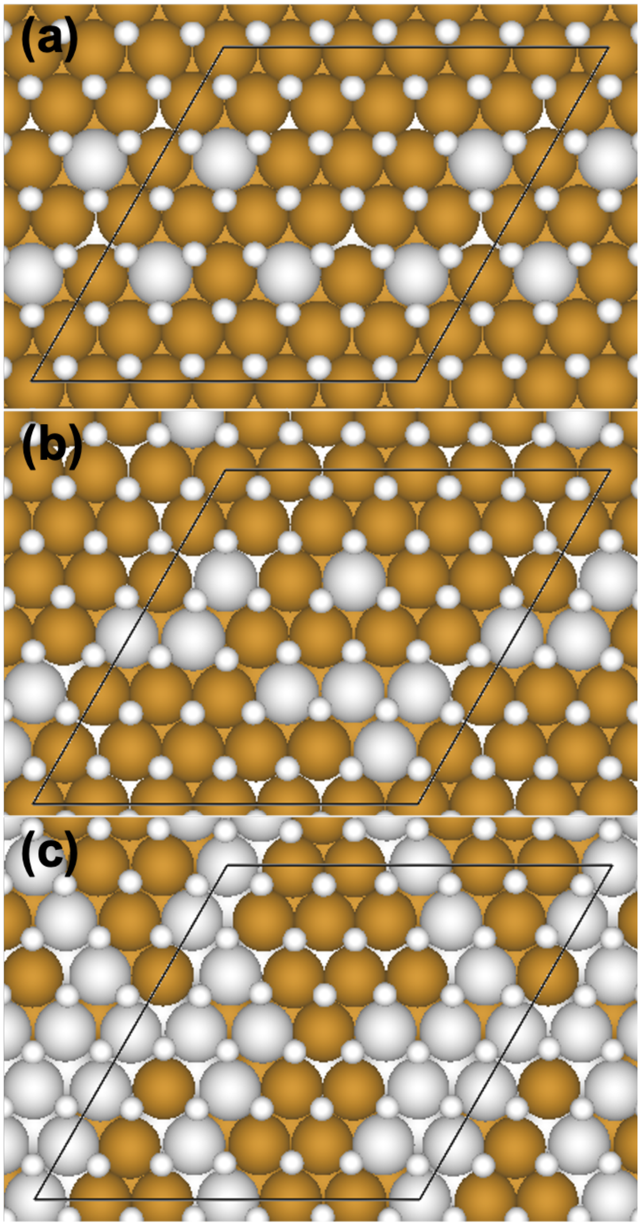

Figure S1: Top views of DFT-optimized 1 ML of H atoms adsorbed on the (6×6) (111) surfaces of: (a) $Pt_{0.15}Fe_{0.85}$, (b) $Pt_{0.25}Fe_{0.75}$, and (c) $Pt_{0.50}Fe_{0.50}$. The surface unit cells are outlined. Color code: Pt = large light spheres; Fe = large dark spheres; H = small light spheres.

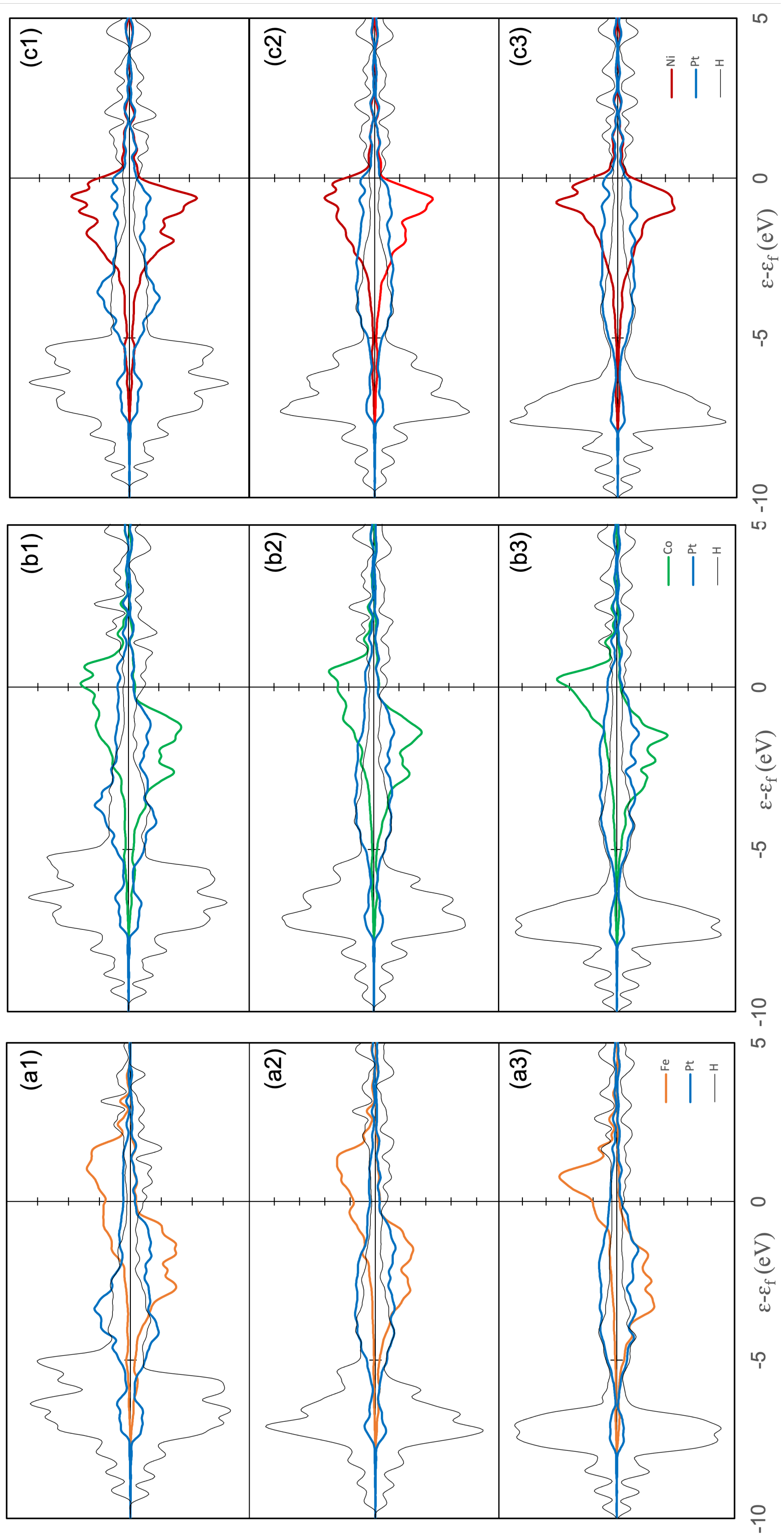

Figure S2: Project density of states (PDOS) for 1 ML of H adsorbed on the (111) facets of (a1-a3): $Pt_{0.25}Fe_{0.75}$, $Pt_{0.50}Fe_{0.50}$, $Pt_{0.75}Fe_{0.25}$; (b1-b3): $Pt_{0.25}Co_{0.75}$, $Pt_{0.50}Co_{0.50}$, $Pt_{0.75}Co_{0.25}$; (c1-c3): $Pt_{0.25}Ni_{0.75}$, $Pt_{0.50}Ni_{0.50}$, $Pt_{0.75}Ni_{0.25}$. Only the d states of those metal atoms in the surface layer (normalized by the number of atoms of each element) and the s states of the H atoms (not normalized) are shown.

All DFT-optimized bulk and surface structures are available upon request.
